# Establishment and characterization of a novel highly aggressive gallbladder cancer cell line, TJ-GBC2

**DOI:** 10.1186/s12935-017-0388-8

**Published:** 2017-02-08

**Authors:** Zhong-Yan Liu, Guo-Li Xu, Hui-Hong Tao, Yao-Qin Yang, Yue-Zu Fan

**Affiliations:** 1 Department of General Surgery, Tongji Hospital, Tongji University School of Medicine, Tongji University, Shanghai, 200065 People’s Republic of China; 2 Laboratory of Tumor Cytology, Tongji University School of Medicine, Tongji University, Shanghai, 200092 People’s Republic of China

**Keywords:** Gallbladder neoplasm, Cell line, Cell culture, Metastasis

## Abstract

**Background:**

Human gallbladder cancer (GBC) is an aggressive malignant neoplasm with a poor prognosis. The development of ideal tools for example tumor cell lines for investigating biological behavior, metastatic mechanism and potential treatment in GBCs is essential. In present study, we established and characterized a GBC cell line derived from primary tumor.

**Methods:**

Primary culture method was used to establish this cell line from a primary GBC. Light and electron microscopes, flow cytometry, chromosome analysis, heterotransplantation and immunohistochemistry were used to characterize the epidemic tumor characteristics and phenotypes of this cell line.

**Results:**

A novel GBC cell line, named TJ-GBC2, was successfully established from primary GBC. This cell line had characteristic epithelial tumor morphology and phenotypes in consistent with primary GBC, such as polygon and irregular cell shape, increased CA19-9 and AFP levels, and positive expression of CK7, CK8, CK19 and E-cadherin with negative vimentin. Moreover, about 25% of the cells were in the S-G2/M phase; abnormity in structure and number of chromosome with a peak number of 90–105 and 80% hypertetraploid were observed. Furthermore, this cell line had higher invasion and highest migration abilities compared to other GBC cell lines; and metastatic-related marker MMP9 and nm23 were positively expressed.

**Conclusions:**

A novel highly aggressive GBC cell line TJ-GBC2 was successfully established from primary GBC. TJ-GBC2 cell line may be efficient tool for further investigating the biological behaviors, metastatic mechanism and potential targeted therapy of human GBC.

## Background

Human gallbladder cancer (GBC) is the most common malignancy of the biliary tract and the leading cause of cancer-related deaths in China, and is a lethal aggressive malignant neoplasm with special malignant biological characteristics, high early local invasion, extensive liver and lymph node metastases, low surgical resection rate (about 10% of GBC patients have a chance to receive surgery in the early stage), high postoperative recurrence rate, less sensitive to chemoradiotherapy, and unfavorable survival [1–3]. Despite imaging technology progress in improving early diagnosis in GBC, prognosis of the patients, who received surgery, chemotherapy and/or radiotherapy, is still not satisfactory [1–4]. Therefore, further studying the special biological behaviors, metastatic and recurrent mechanisms, and potential interventions of GBCs is of special significant, and remain challenging [5–7]; and novel GBC cell lines as ideal study models in vitro and in vivo are urgently developed. However, the establishment of highly aggressive GBC cell lines derived from primary tumor is very few and not thoroughly elucidated [8–24]. In present study, we established a novel highly aggressive GBC cell line derived from primary GBC, TJ-GBC2, which may prove to be an efficient tool for further investigation of the metastatic mechanism and potential treatment of this malignant disease.

## Methods

### Original tumor

This study was carried out in accordance with the Declaration of Helsinki and the official recommendations of Chinese Community Guidelines, and was approved by the Ethics Committee and the Institutional Review Board at the Tongji Hospital. Written informed consent was obtained from this patient and his relation.

**Fig. 1:**
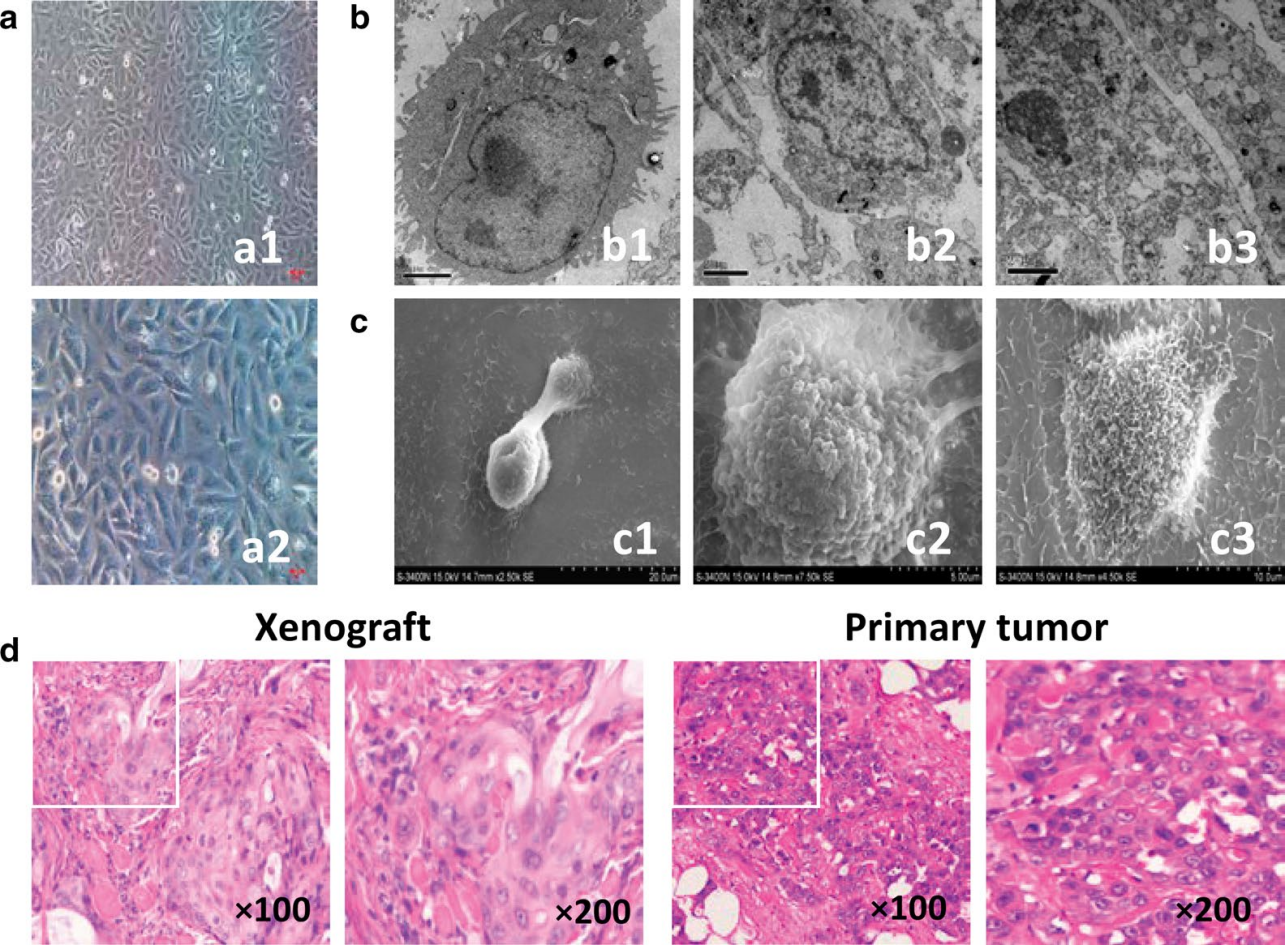
Epithelial tumor morphological characteristics of TJ-GBC2 cell line. **a** Morphology of TJ-GBC2 cell lines (at the passage 35–50) under a light microscopy (a1 × 100, a2 × 200). Cells grew mainly in clusters of polygonal cells, partially fusiform, spindly or irregular shape as an adherent monolayer sheet with characteristic epithelial cell morphology, in addition to big nucleoplasm ratio and multiple nucleoli. **b** Karyomegaly, dicaryon, clear cellular organelle structures such as ribosomes, mitochondria, prosperous endoplasmic reticulum, Golgi apparatus and secretory granules in cytoplasm, and lots of microvilli outside the network and cell connection were clearly observed under a transmission electron microscopy (TEM; b1–b3, × 10,000). **c** The divided cell and its surface full of densely filamentous microvilli and lamellar prominences were clearly visualized under a scanning electron microscopy (SEM; c1 × 2500, c2 × 7500, c3 × 4500). **d** The xenograft of TJ-GBC2 cells in nude mice in vivo presented typical GBC features in nest-streak like arrangement with atypical hyperplasia, caryokinesis and poor differentiation e.g. most of mucous epidermoid carcinoma differentiation, which were consistent with primary tumor of GBC

A 67-year-old Chinese man with symptoms of acute cholecystitis was referred to our hospital. High levels of CA19-9 (>1000 U/ml), CA242 (58.4 U/ml), CA50 (428.4 U/ml) and CEA (7.8 ng/ml) were detected in the patient’s serum by radioimmunoassay, whereas serum AFP showed in a normal range. Abdominal CT revealed a thickened, irregular gallbladder wall (1.5 cm) with involvement of the liver bed (6.0 cm) and hepatic bile duct dilatation. A radical GBC resection with partial hepatectomy was done. The postoperative pathological examination of the en bloc resected specimen showed that the GBC represented a poor differentiated adenocarcinoma forming nest-streak like arranged structures with atypical hyperplasia and caryokinesis, and most cells were of mucous epidermoid carcinoma differentiation (Fig. 1). The patient died about seven and a half months after operation with tumor recurrence, liver and extrahepatic bile duct metastases, and jaundice and hepatic failure.

### Primary culture in vitro

The cell line was established from a primary tumor, which was surgically obtained from above GBC patient. After rinsing thrice with sterile PBS containing antibiotics, the tumor was minced into small fragment having a diameter of 1 mm using a scalpel, and completely eliminated subcutaneous fat and submucosa. The fragment was rinsed with PBS for 3 min, wet with 20% FBS (Corning, USA), then seeded into 25 ml culture bottle (Costar, USA). And, the culture bottle was inversionally incubated in a humidified incubator (SANYO, Japan) at 37 °C in a 5% CO_2_ atmosphere for 4 h, then was put in normal direction, and added 3–4 ml DMEM/F12 (Gibco, USA) containing 20% FBS and 100 U/ml antibiotics along the edge of culture bottle slowly. After 5-day incubating, a small amount of cells climbing out around the tissue fragment, and a large number of lymphocytes and other miscellaneous cells were observed. The growth medium was renewed and replaced every 3 days, and the bottles were regularly checked for epithelial cells and fibroblast outgrowth. If fibroblast growth was observed during primary cultures, differential trypsinisation was used to obtain a pure tumor-cell population. After 5–6 passages tumor cells were basically purified. The cell line was cultured for >60 passages.

### Heterotransplantation in vivo

This study was carried out in accordance with ARRIVE (Animal Research: Reporting of In Vivo Experiments) guidelines [25], and was approved by the Ethics Committee of Animal Experiments and the Institutional Review Board at the Tongji Hospital. TJ-GBC2 cells at the passage 35 were used to determine their tumorigenicity in nude mice. The cultured cells (1 × 10^7^/ml) were harvested, washed, suspended in 0.1 ml of PBS, were then injected subcutaneously into the right flanks of 4-week-old athymic female nude mice (Balb/c-nu; Shanghai Silaike). Animals were examined every week for the development of tumors. Tumor-bearing mice were sacrificed. And, tumor tissue was excised, fixed in 10% formalin, and processed for histopathology and immunohistochemistry.

### Morphologic observation in vitro and in vivo

Morphologic observation included morphologic structure and ultrastructure of TJ-GBC2 cells in vitro and morphologic structure of the xenograft of TJ-GBC2 cell lines in nude mice in vivo. For microscopy, the cultured TJ-GBC2 cells were photographed directly without staining, and histomorphologic structure of the xenograft in vivo was observed with H&E staining under a phase contrast microscope (Caikang XDS-100, Shanghai, China). For electron microscopy, the monolayer cells cultured in the flasks were fixed with 2.5% glutaraldehyde in 1 ml PBS (pH7.2), and post-fixed in a solution of 1% osmium tetraoxide. After dehydration in graded ethanol, the samples were then embedded in Epon resin. Ultrathin sections were stained with 2.3% uranyl acetate and lead citrate, and examined under a TEM (Jeol-1230) or SEM (Hitachi S-3400 N, Japan).

### Cell proliferation, cell cycle assays and chromosome analysis in vitro

Cultured TJ-GBC2 cells (experimental group) and SGC996 cells derived from another primary GBC (control group) were used in this experiment. Cells were grown in a 96-well plate (5 × 10^4^ cells/100 μl/well) in DMEM/F12 medium with 10% FBS. The cell numbers were measured by a MTT assay according to the protocol provided by the MTT manufacturer. The doubling times were determined from the growth curve.

Cell cycle analysis was performed using a FCM (FlowJo software). Cells (1 × 10^6^) in an exponential growth phase were harvested and fixed with cold 70% alcohol after rinsing with cold PBS twice, incubated at 4 °C environment for 24 h. After being centrifuged at 1000 r/min for 5 min, the cells were rinsed with cold PBS once, suspended in 500 μl PBS with 5 μl RNAase (10 mg/ml; Invitrogen, USA) and incubated at 37 °C for 30 min, then stained with 5 μl propidium iodide (5 mg/ml; Invitrogen). This cell cycle analysis was performed in triplicate.

Chromosome analysis was performed for cells at the passage 50–54. Cells in an exponential growth phase were karyotyped using a standard air-dried method after treatment with a final concentration of 0.01 μg/ml colcemid for 2 h. A total of 50 metaphase spreads were counted to determine the modal number.

### Invasion and migration assays in vitro

Five human GBC cell lines including TJ-GBC2, GBC-SD, NOZ, OCUG-1 and SGC996 were used to evaluate the migration and invasive abilities of GBC cells. TJ-GBC2 cell line were maintained in DMEM/F12 supplemented with 10–20% FBS; GBC-SD (Type Culture Collection of the Chinese Academy of Sciences, Shanghai, China) and NOZ (gifted from Professor Liu YB) cell lines were maintained in DMEM (Corning, USA) supplemented with 10% FBS; whereas the OCUG-1 (gifted from Professor Liu YB) and SGC-996 (Laboratory of Tumor Cytology, Tongji University School of Medicine, Shanghai, China) cell lines were maintained in RPMI-1640 medium (Gibco, USA) supplemented with 10% FBS, respectively.

Cell invasion in vitro was assessed using the Transwell chambers (Corning, USA). 200 μl cell suspensions (5 × 10^4^/well) were seeded onto the upper chamber, 600 μl fresh growth medium with 10% FBS were placed into the lower chamber. After 24-h in a humidified incubator at 37 °C with 5% CO_2_, cells that invaded through the basement membrane were stained with Giemsa (Sigma, USA), and counted under an inverted light microscope (Caikang XDS-100) in 5 independent fields at ×200 magnification. Three independent experiments were performed.

Cell migration in vitro was determined using a wound-healing assay. 200 μl cell suspensions (5 × 10^5^/well) were seeded in a 96-well plate (VP scientific, USA) for 24 h. When cultured cells reached 50% confluence in a single layer, a wound was scratched at the center of the cell monolayer using a sterile scratch tester. Then, cells were washed with sterile PBS to remove floating cellular debris, and added with growth medium with FBS for 24 h. The cell migrating area was scanned and analyzed at 0 h, 8 h and 24 h using a Cellomocs (Thermo, USA), and was observed under an inverted light microscope (Caikang XDS-100) at × 50 magnifications. Cell migration area (pixel area) = (S3 + S4) − (S1 + S2). All experiments were performed in triplicate.

### Epithelial tumor marker and metastatic marker assays in vitro and in vivo

Epithelial tumor markers including CEA, CA19-9 and AFP in the supernatant from the cell culture were detected using an electrochemistry luminescence immunity analyzer (Cobas E601, Roche, USA). The cultured cells (1 × 10^5^) were collected and centrifuged at 1000 r/min for 5 min. The supernatant was collected for CEA, CA19-9 and AFP. Pure growth medium was selected for a negative control.

Epithelial markers including CK7, CK8, CK19 and E-cadherin, and mesenchymal marker vimentin, tumor marker p53, and metastatic marker nm23 and MMP9 proteins from the sections of primary GBC and tumor xenograft of nude mice were examined using immunohistochemistry SABC method. The sections (4-μm) were dehydrated in xylene and graded ethanol series, were added in order with primary antibody (CK7, CK8, CK19, E-cadherin, vimentin, p53, nm23 or MMP9; all 1:100, rabbit monoclonal antibody), biotinylated secondary antibody, SABC reagents and DAB solution (all from Santa Cruz, USA), respectively; i.e., the samples were stained with Santa Cruz ABC kit according to the protocol provided by the manufacturer, and observed under an optical microscope (Olympus IX70, Japan) with ×100–400 objectives. Light brown or tan particles in cytoplast were regarded as positive. For negative control, the slides were treated with PBS in place of primary antibody.

### Statistical analysis

All data were expressed as mean ± SD and analyzed using SPSS (22.0 version software, IBM, USA). Statistical analyses to determine significance were tested with Student’s *t* test and F test. *P* < 0.05 was considered statistically significant.

## Results

### A novel GBC cell line, TJ-GBC2

This present study, a cell line was in vitro successfully established from a primary tumor, which was derived from a surgically resected specimen of primary GBC, using primary culture of tissue fragment and differential adherent purified method; and the cell line was successfully frozen, resuscitated and cultured in DMEM/F12 medium supplemented with 10–20% FBS for >60 generations. In June 1999, our Tongji University established first human GBC cell line SGC-996, which was derived from primary GBC. Thus, this novel GBC cell line is currently denominated as TJ-GBC2 (Tongji Hospital, Tongji University School of Medicine; Gallbladder Cancer-2).

### Epithelial tumor morphological characteristics of TJ-GBC2 cell line

Here, the epithelial tumor morphological characteristics of the TJ-GBC2 cells in vitro and the xenograft of TJ-GBC2 in nude mice in vivo were observed, and compared with the morphological characteristic of primary GBC. As showed in Fig. 1, TJ-GBC2 cells (the passage 35 and 50) grew mainly in clusters of polygonal cells, partially fusiform, spindly or irregular shape as an adherent monolayer sheet with characteristic epithelial cell morphology, in addition to big nucleoplasm ratio and multiple nucleoli (Fig. 1a). Moreover, karyomegaly, dicaryon, and clear cellular organelle structures such as abundant ribosome, mitochondria, prosperous endoplasmic reticulum, Golgi apparatus and secretory granules in cytoplasm, lots of microvilli outside the network and cell junctions between tumor cells (Fig. 1b), and the divided cell and its surface full of densely filamentous microvilli and lamellar prominences (Fig. 1c) in accord with epithelial cell morphology were clearly visualized under a TEM or SEM. Furthermore, in vivo xenograft in nude mice presented typical GBC features in nest-streak like arrangement with atypical hyperplasia, caryokinesis and poor differentiation e.g. most of mucous epidermoid carcinoma differentiation, which were consistent with primary tumor of GBC (Fig. 1d).

### Growth characteristics of TJ-GBC2 cell line in vitro and in vivo

**Fig. 2:**
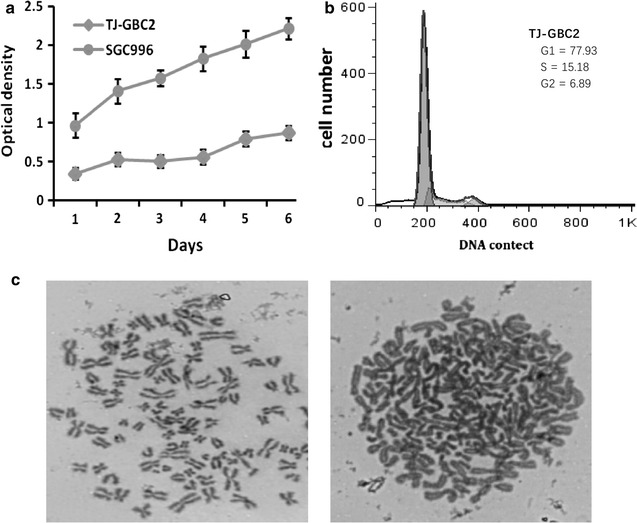
Proliferation-related characteristics and karyotype of TJ-GBC2 cell line. **a** The growth curve of TJ-GBC2 and SGC996 assayed using a MTT method. TJ-GBC2 cell line has a less vigorous growth tendency compared to SGC996 in vitro. **b** Cell cycle of TJ-GBC2 cell line detected by FCM, about 25% of the cells were in the S-G2/M phase. **c** Karyotype analysis of TJ-GBC2 cell line at the passage 50 (oil-immersion lens, ×1000): the number of chromosomes ranged from 48 to 132, with a peak number between 90 and 105, 80% of which is hypertetraploid

Growth characteristics of TJ-GBC2 cell line composed of the proliferation-related properties including proliferation capability, cell cycle and karyotype of TJ-GBC2 cells in vitro and the tumor growth of xenograft e.g. heterotransplantation in vivo. The proliferation capability of TJ-GBC2 cells was assayed using the MTT method. Cell growth curve of TJ-GBC2 cell line was showed in Fig. 2a, i.e. TJ-GBC2 cell line has a less vigorous growth tendency compared to SGC996 in vitro. Moreover, the cell cycle of TJ-GBC2 cell line analyzed using FCM was found that about 25% of the cells were in the S-G2/M phase (Fig. 2b). Further, complicated karyotype and abnormal chromosome number of TJ-GBC2 cell line was revealed using chromosome analysis, which included gains, losses, translocations and other abnormalities of karyotype; and the number of chromosomes ranged between from 52 to 132, with a peak number between 90 and 105, 80% of which is hypertetraploid (Fig. 2c). Furthermore, tumor growth of xenograft in vivo was observed. 2–4 weeks after TJ-GBC2 cells were injected subcutaneously into the right flanks of nude mice, a visible subcutaneous xenograft with a slight slower growth rate was found; at 8 weeks, xenograft at diameter of range 0.4 cm–0.5 cm were observed in all (8/8, 100%) mice.

### Epithelial tumor characteristics of TJ-GBC2 cell line

**Fig. 3:**
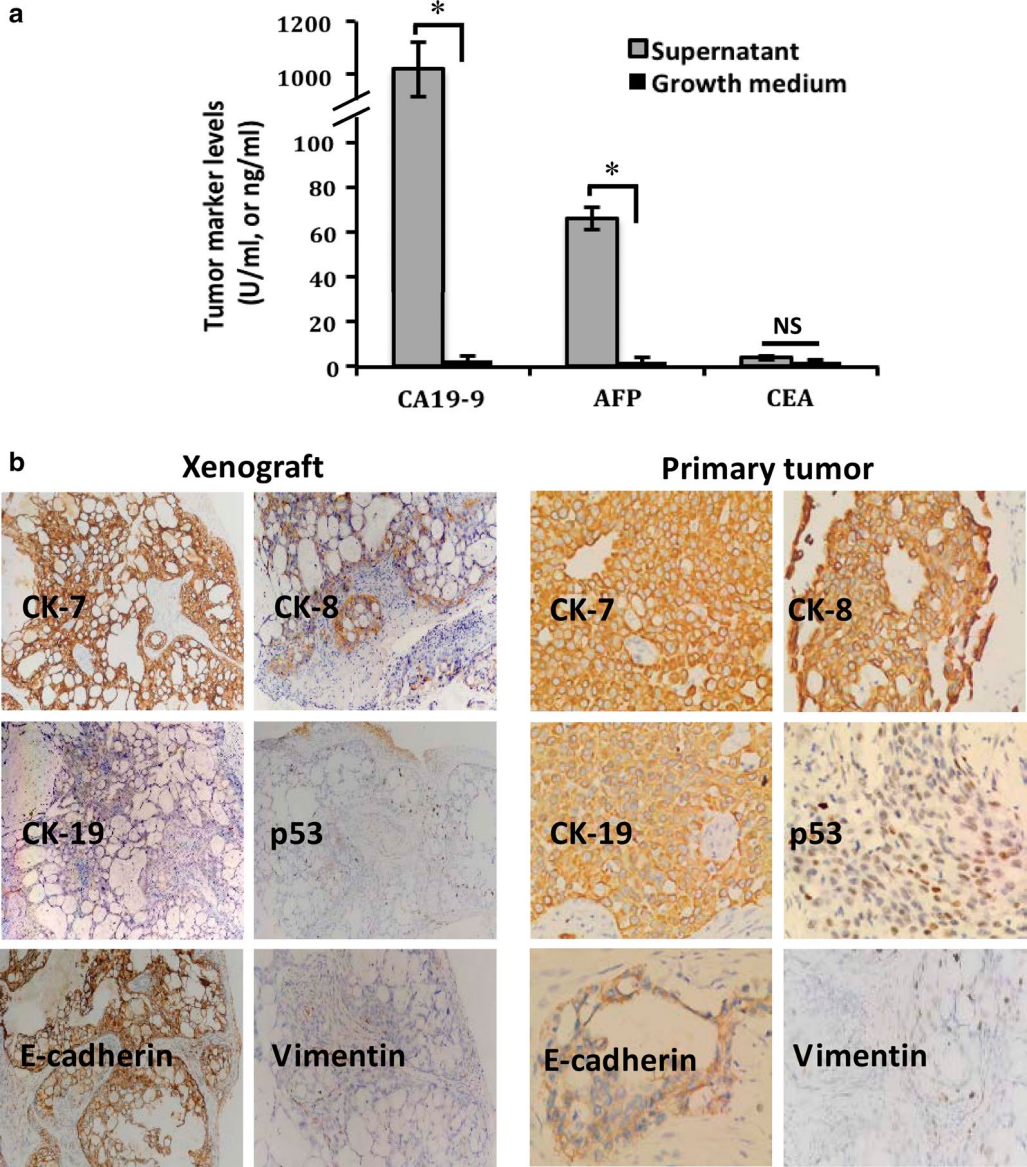
High expression of epithelial or epithelial tumor marker of TJ-GBC2 cell line in vitro and in vivo. **a** Epithelial tumor markers of the culture supernatant of TJ-GBC2 cells in vitro. Supernatant CA19-9 (>1000 vs. 2.86 U/ml, normal value: <39 U/ml) and AFP (65.85 vs. 0.20 ng/ml, normal value: <4.7 n/ml) levels were higher than those of pure growth medium (all *P* = 0.000); but CEA showed normal levels (0.61 vs. 0.61 ng/ml, *P* > 0.05; normal value: <7 ng/ml). **b** Expression of characteristic epithelial and mesenchymal cell markers in the xenografts of nude mice in vivo. Epithelial marker CK7, CK8, CK19 and E-cadherin were positively expressed in the xenografts, with positive p53 expression in few cells and negative mesenchymal marker vimentin expression, which is in accord with the results of primary tumor of human GBC

In order to testify whether TJ-GBC2 cell line has epithelial tumor characteristics, we further detected epithelial tumor markers of the culture supernatant of TJ-GBC2 cells in vitro and characteristic epithelial and mesenchymal cell markers of the xenografts in vivo, and compared these markers with primary GBC expressed markers. As showed in Fig. 3a, epithelial tumor marker CA19-9 (>1000 vs. 2.86 U/ml, normal value: <39 U/ml) and AFP (65.85 vs. 0.20 ng/ml, normal value: <4.7 n/ml) levels in the culture supernatant were higher than those of pure growth medium (all *P* = 0.000); but CEA showed normal levels (0.61 vs. 0.61 ng/ml, *P* > 0.05; normal value: <7 ng/ml). Characteristic epithelial marker CK7, CK8, CK19 and E-cadherin were positively expressed in the xenografts of nude mice, with positive p53 expression in few cells and negative mesenchymal marker vimentin expression (Fig. 3b), which is in accord with the results of primary tumor. Took together, these results verified TJ-GBC2 is an epithelial tumor original cell line.

### Highly aggressive characteristic of TJ-GBC2 cell line

**Fig. 4:**
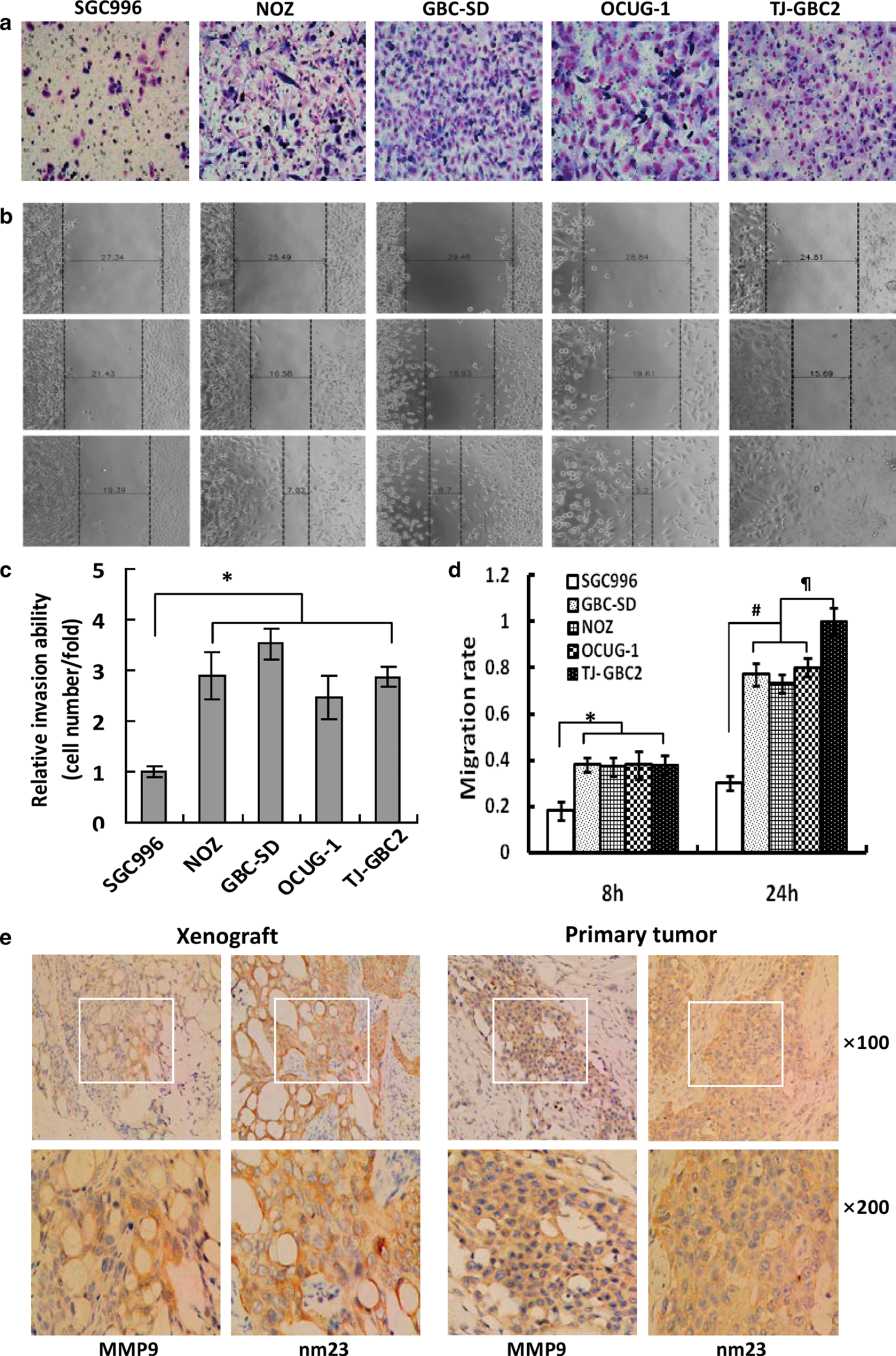
The highly aggressive characteristic of TJ-GBC2 cell line. **a**, **c** The invasion capability of five GBC cell lines in vitro (Transwell invasion assay; Giemsa stain, ×200). The number of TJ-GBC2 cells that invaded through the basement membrane was significantly more than that of SGC996 (*P < 0.000), without difference on the number of invaded cells among NOZ, GBC-SD, OCUG-1, TJ-GBC2 cell lines (all *P* > 0.05). **b**, **d** The migration capability of five GBC cell lines in vitro (Wound healing assay). The migration rate of TJ-GBC2, GBC-SD, NOZ and OCUG-1 cell lines was significantly stronger than that of SGC996 (*P < 0.001, ^#^ *P* < 0.001); of them, TJ-GBC2 cell line had a strongest migration ability compared to GBC-SD, NOZ and OCUG-1 cell lines (all ^¶^ *P* < 0.01). **e** The expression of metastatic-related marker MMP9 and nm23 protein in the xenograft of nude mice and primary tumor of human GBC. MMP9 and nm23 protein was positively expressed in the xenograft of nude mice, which is consistent with the result of primary tumor of human GBC

In order to identify the aggressive capability of TJ-GBC2 cell line, the invasion and migration assays for TJ-GBC2 cell line were performed; and, the two GBC cell lines derived from primary GBC: GBC-SD and SGC996, and two GBC cell lines derived from ascites of GBC patients: NOZ and OCUG-1 were selected for positive controls. As showed in Fig. 4, the number of TJ-GBC2 cells that invaded through the basement membrane, i.e. invasion ability was significantly more than that of SGC996 (>double cell number/fold, *P < 0.000); whereas no difference on the number of invaded cells among NOZ, GBC-SD, OCUG-1 and TJ-GBC2 cell lines was observed (all *P* > 0.05; Fig. 4a, c). Moreover, the migration ability of GBC cell lines was assayed using a wound-healing assay. The result showed that the relative migration rate of TJ-GBC2, GBC-SD, NOZ and OCUG-1 cell lines for 8 and 24 h was significantly higher than that of SGC996 (*P < 0.05, ^#^ *P* < 0.01); of them, TJ-GBC2 cell line had a highest migration ability compared to GBC-SD, NOZ and OCUG-1 cell lines (all ^¶^ *P* < 0.01; Fig. 4b, d), and after 24 h in TJ-GBC2 cell line group the scratched wound of the cells completely healed. In order to verify the aggressive and metastatic capabilities of TJ-GBC2 cell line, we further examined the expression of metastatic-related marker MMP9 and nm23 in the xenograft of nude mice. The result showed that MMP9 and nm23 were all strong positively stained in xenograft of nude mice, which is consistent with result of primary human GBCs (Fig. 4e). Therefore, TJ-GBC2 cell line was identified as a highly aggressive GBC cell line.

## Discussion

Human GBC is a highly aggressive malignant tumor with special biological behavior and poor prognosis. Surgical resection, chemotherapy and radiotherapy for the disease are disappointing [1–6]. So, the development of novel adjuvant therapies, potential anticancer agents or molecularly targeted therapeutics for human GBC on the base of comprehensive investigating the biological behaviors and metastatic mechanism are very necessary; and novel GBC cell lines which were used as ideal experimental models in vitro and in vivo are urgently developed. In present study, we firstly established a novel highly aggressive GBC cell line derived from primary tumor, TJ-GBC2.

Human GBC cell lines are relatively scarce. Nowadays, more than a dozen of GBC cell lines were available, including G-415, GBK-1, KMG-A, FU-GBC-1, FU-GBC-2, NOZ, PTHrP-GBK, GB-d1, TGBC1TKB, TGBC2TKB, OCUG-1, TYGBK-1, HAG-1, GBC-SD, SGC-996, EH-GB1 and EH-GB2 [8–24]. Of these, most were derived from the metastatic lesions of GBC patients, such as NOZ, OCUG-1, FU-GBC-2 and EH-GB1 from the ascites or the abdominal wall [11, 13, 21], TYGBK-1 from a lymph node [24], and EH-GB2 from liver metastatic site [23]; some were derived from primitive cultured tumor that planted in nude mice [18]; whereas others had themselves features, for example, GBK-1 was derived from human colony stimulating factor-producing GBC [9], KMG-A from AFP-producing GBC [10], and PTHrP-GBK from parathyroid hormone-related peptide producing GBC [16]. Indeed, it is much more difficult to generate a primary cultured GBC cell line from a primary tumor than from metastases and ascites. This is because there are more fibrous tissues in GBC lesions, and biliary obstruction and infection contaminated GBC specimens. It is well known that the cell lines derived from ascites or other metastatic sites, or primitive cultured xenograft in nude mice were at least limited in two respects: one limitation was these cell lines derived from metastatic site losing the properties possessed in primary tumor, and cell line monoclonality that could not reflect heterogenic properties of the pleomorphic type of GBC; another limitation was these cell lines derived from xenografts of nude mice with a part of the immune function still having stronger immune-resistance. Therefore, culture of primary tumor of GBC may be a better way to build a cell line so as to accurately reflect the characteristics of the primary tumor cells [26]. In present study, we successfully established a novel GBC cell line (TJ-GBC2) from a Chinese patient with primary GBC, with retaining characteristic epithelial tumor morphology and phenotypic in consistent with primary GBC. Although the cell line appears to have no prominent capacities of proliferation and growth in vitro and in vivo, chromosome analysis presented abnormity in structure and number of chromosome, and most cells (about 80%) were hypertetraploid, which implied high malignant potential. Coexistence of polygon, fusiform, irregular shape cells further implied that the cell line was derived from multicenter or polyclone. Therefore, primary cultured TJ-GBC2 cell line derived from primary tumor of GBC may reflect more accurately the characteristics of the primary GBC cells. TJ-GBC2 cell line proved to be an efficient tool for further investigation of the metastatic mechanism and potential targeted therapy of human GBC.

Epithelial tumor characteristics include epithelial morphological features, positive expression of epithelial markers with negative mesenchymal expression, and positive expression of epithelial tumor markers. As showed in Figs. 1 and 3, TJ-GBC2 cell line has characteristic epithelial tumor phenotypes as well as above characteristic epithelial tumor morphology in consistent with primary GBC. As we know, CK7, CK8, CK19 and E-cadherin are special epithelial markers; vimentin is characteristic mesenchymal marker; whereas CA19-9 and AFP are epithelial tumor markers. Electrochemistry luminescence immunity analysis showed that levels of CA19-9 and AFP were significantly increased in the culture supernatant of TJ-GBC2 cells; that CK7, CK8, CK19 and E-cadherin proteins were positively expressed in the xenograft of nude mice, with negative expression of mesenchymal marker vimentin, which is in accord with the results of primary tumor. These results verified TJ-GBC2 is an epithelial original cell line.

Metastasis, the spread of malignant cells from a primary tumor to distant sites and forms a tumor of same nature [27, 28], is the biggest problem to cancer treatment [29]. Abilities of migration and aggression affect the invasion and metastasis of tumor cells to a large extent. Intractability of gallbladder cancer also attribute to its early invasion and metastasis. In present study, we detected the aggressive and migration capabilities of five GBC cell lines, and expression of metastatic-related marker nm23 and MMP9 in the xenograft of TJ-GBC2 cell lines in nude mice. The results showed that TJ-GBC2 cell line had higher invasion ability and the highest migration ability compared to other human GBC cell lines such as GBC-SD and NOZ; and that MMP9 and nm23 were positively expressed in xenograft of nude mice, which is consistent with result of primary tumor of human GBCs. It was reported that GBC-SD is so far a human GBC cell line having the highest aggressive capability, which was derived from primary GBC [15, 20, 22]; whereas NOZ is a human GBC cell line having the highest aggressive capability, which was derived from metastatic site of primary GBC [11, 30]. In this study, cells that invaded through the basement membrane for 24 h having more than double cell number/fold in invasive assay and cells that were in a wound healing experiment for 24 h having completely healed cell wound were was used to defined as highly aggressive cell line. Considering GBC-SD and NOZ as the highest aggressive capability GBC cell lines, TJ-GBC2 cell line having a higher invasion ability and the highest migration ability compared to GBC-SD, NOZ, OCUG-1 and SGC-996, and positive expression of metastatic-related marker MMP9 and nm23 in the xenograft of TJ-GBC2 cells in nude mice, which is consistent with result of primary GBC, we thus identified TJ-GBC2 as a highly aggressive GBC cell line.

## Conclusions

Collectively, in this study, we firstly established a novel highly aggressive TJ-GBC2 cell line derived from a Chinese patient with primary GBC. This GBC cell line has characteristic epithelial tumor morphology and phenotypes in consistent with primary GBC and highly aggressive potential, and reflects more accurately the characteristics of the primary GBC cells. Thus, TJ-GBC2 cell line may provide an efficient tool for further investigating the metastasis mechanism, early diagnosis and potential targeted therapy of human GBC.

## Abbreviations

GBC: gallbladder cancer
PBS: phosphate-buffered saline
FBS: fetal calf serum
DMEM/F12: Dulbecco’s modified Eagle’s medium/F12
TEM: transmission electron microscopy
SEM: scanning electron microscopy
MTT: tetrazolium-based colorimetric assay
CT: computed tomography
FCM: flow cytometry
CEA: carcinoembryonic antigen
CA19-9, CA242, CA50: carbohydrate antigen 19-9, -242, -50
AFP: α-fetoprotein
CK7, CK8, CK19: cell keratin-7, -8, -19
MMP9: matrix metalloproteinase-9
H&E: hematoxylin and eosin
SABC: strept avidin-biotin complex
DAB: 3,3′-diaminobenzidine
